# Association of Structural Fires in New York City With Inequities in Safe Heating for Immigrant Communities

**DOI:** 10.1001/jamanetworkopen.2023.1575

**Published:** 2023-03-03

**Authors:** Eloise Stanton, Julia Biedry, Danielle Rochlin, Clifford C. Sheckter

**Affiliations:** 1 Department of Surgery, University of Southern California, Los Angeles; 2 Tableau Software, Salesforce, Mountain View, California; 3 Memorial Sloan Kettering Cancer Center, New York, New York; 4 Department of Surgery, Stanford University, Palo Alto, California; 5 Regional Burn Center, Santa Clara Valley Medical Center, San Jose, California

## Abstract

This cross-sectional study assesses the association of heating complaints with structural fires in New York, New York.

## Introduction

On January 9, 2022, a structural fire claimed the lives of 8 children and 9 adults in the Bronx borough of New York, New York.^1^ The predominantly Gambian immigrant community experiences impoverished conditions that include apartment buildings without reliable heating, forcing many to use more dangerous alternatives, such as space heaters or lighting fires. Reports suggest that in low-income, historically Black and Latinx communities, heating complaints are often ignored.^2^ While this pattern implies housing inequities, landlord negligence, and greater risk of residential fires in low socioeconomic neighborhoods, this postulation has not been quantitatively evaluated. We investigated the association between structural fires and heating complaints in New York City, leveraging municipal data from 59 community districts.

## Methods

This cross-sectional study was exempt from approval and informed consent by the Stanford University institutional review board because we used deidentified, publicly available data. This study follows the Strengthening the Reporting of Observational Studies in Epidemiology (STROBE) reporting guideline for cross-sectional studies.

Data from the New York City Open Data Portal Fire Incident Dispatch and Heat/Hot Water Complaints were merged to identify the number of heating complaints and structural fires per month in each community district in New York City from 2017 to 2022. Population and demographic comparisons were made using 2020 decennial US Census data. The primary outcome was number of structural fires per month, modeled with linear mixed-effects multivariable regression. Community districts were treated as random intercepts, given demographic and architectural variation between districts. Statistical analyses were performed using Stata/IC version 17.0 (StataCorp). The frequency of heating complaints and structural fires and the proportion of Black and Latinx residents per community district were geomapped with Tableau software (Tableau Software). Race was classified according to census definitions. Data were analyzed from April through June 2022.

## Results

**Figure 1.**
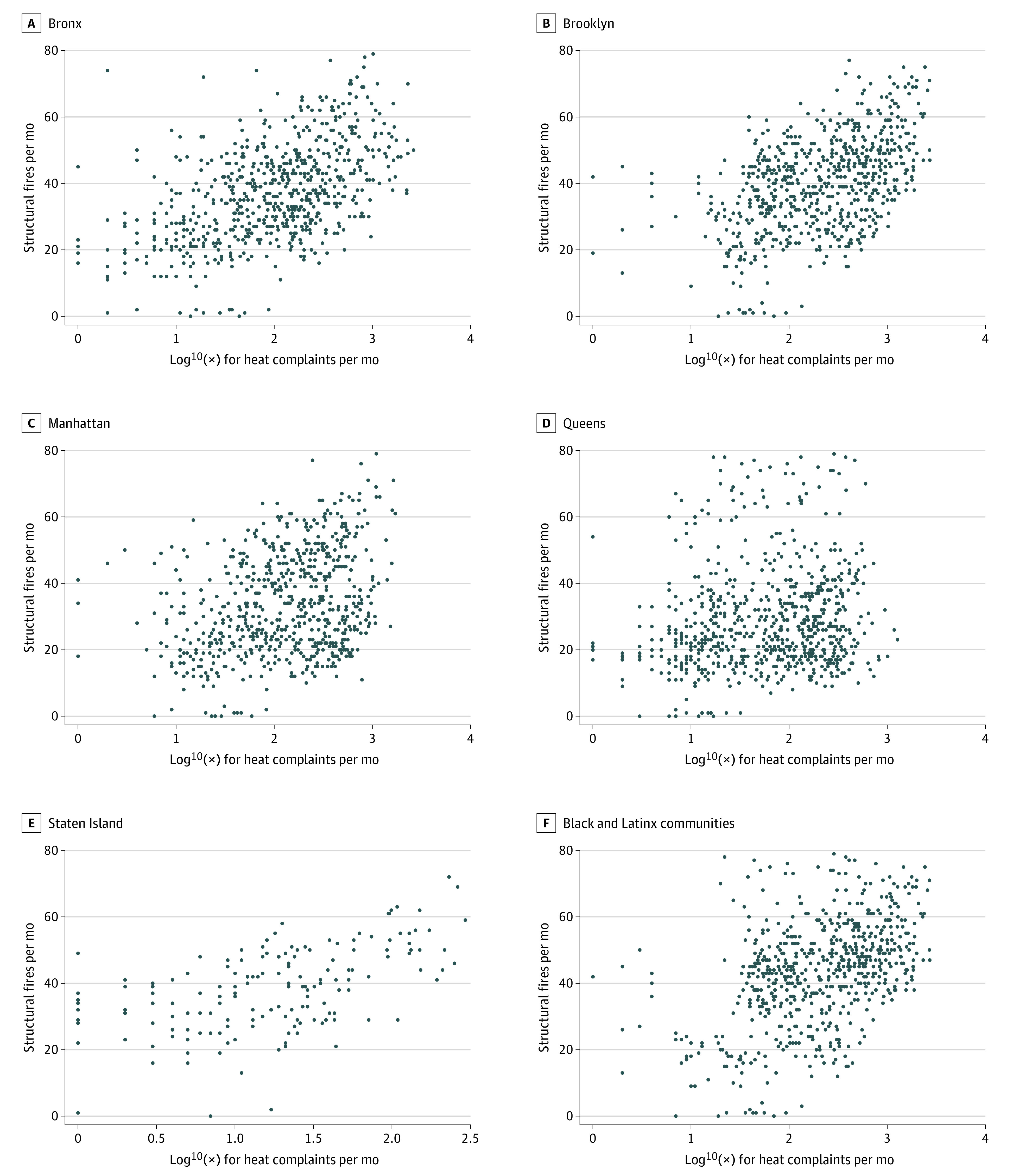
Scatterplots of Monthly Fire Frequency vs Log_10_ of Heat Complaints per Month by Community District

**Figure 2.**
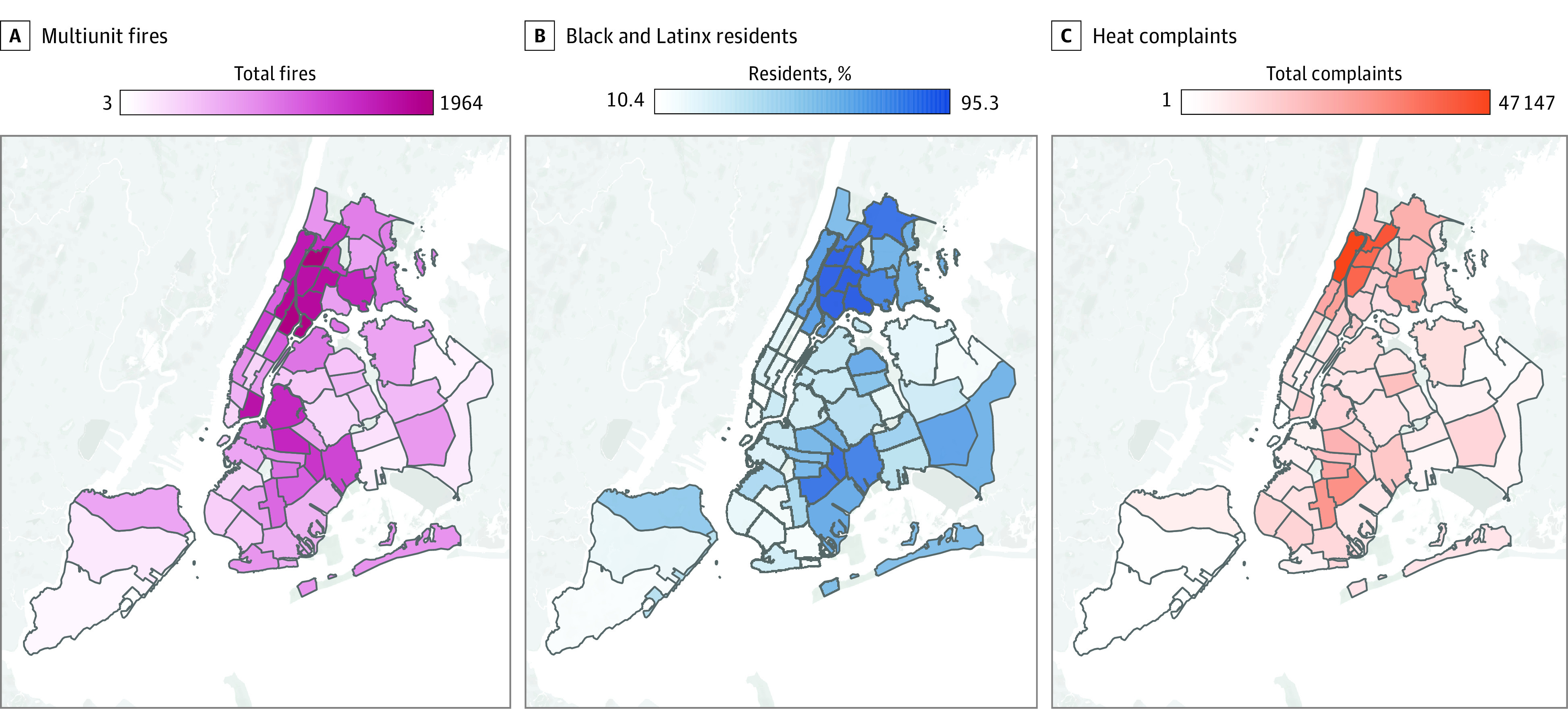
New York, New York Multiunit Residential Fires by Community District, 2017-2022

Within New York City’s 59 community districts, there were 3877 heating complaints and 3989 structural fires from 2017 to 2022. The median (range) number of heat complaints per month was 131 (1-8213) complaints, while the median (range) number of structural fires per month was 30 (0-129) fires (Figure 1). The mixed-effects model found a significant association between structural fires and frequency of heat complaints (β = 0.013; 95% CI, 0.012-0.014; *P* < .001). Furthermore, the model found significant variance among community districts in estimating structural fires (σ^2^ = 215; *P* < .001) (Figure 2). The likelihood ratio test comparing to a simple linear regression yielded a *P* < .001 in favor of the mixed-effects model.

## Discussion

This cross-sectional study found that the frequency of heating complaints was significantly associated with the frequency of structural fires in New York City. Importantly, this association varied across community districts, with more fires occurring in districts with greater proportions of Black and Latinx residents. Our results highlight a pattern adversely affecting marginalized racial and ethnic communities, such as Black and Latinx communities, in which ongoing ignored heating complaints and landlord negligence may lead residents to use unsafe heating practices, inadvertently creating structure fires, morbidity, and death.

Although burn survivorship has increased considerably over the past 50 years due to advances in critical care and surgical treatments, carbon monoxide toxicity and massive body surface area burns still portend high mortality.^3^ Burn prevention remains the hallmark strategy to ameliorate burn-related morbidity and mortality.^4^ The association between heating complaints and fires offers the ability to potentially anticipate future fires. Serial analysis of heating complaints within community districts could trigger investigations to identify involved apartments and engineer safe heating. Knowledge of faulty heating is known in many structures, yet the process of enforcing these violations may not be effective. A proposed New York state law requires that space heaters have automatic shut-off mechanisms when tipped over^5^; however, this is not a replacement for central heating and still places residents at fire risk. Safe heating is a right in most cities within the US, including New York City, and existing laws should be enforced.^6^ Our study is limited by its observational nature; repeating this study in other cities could further validate the association between heating complaints and structure fires. Additionally, for reasons relating to social, cultural, and legal factors, marginalized individuals may be less likely to report heating complaints, which would bias our results towards the null hypothesis.

## Notes

Supplement.
Data Sharing Statement
