# Supplementary material for: Association of Structural Fires in New York City With Inequities in Safe Heating for Immigrant Communities
Source: JAMA Netw Open. 2023 Mar 3;6(3):e231575. doi: 10.1001/jamanetworkopen.2023.1575 (PMC9984975; doi:10.1001/jamanetworkopen.2023.1575)
Supplement: Supplement. — Data Sharing Statement [file jamanetwopen-e231575-s001.pdf]

Data Sharing Statement

Stanton. Association of Structural Fires in New York City With Inequities in Safe Heating for Immigrant Communities. *JAMA Netw Open*. Published March 03, 2023.
doi:10.1001/jamanetworkopen.2023.1575

Data
Data available: No

Additional Information
Explanation for why data not available: These are publicly available data that anyone can access from New York City websites.
